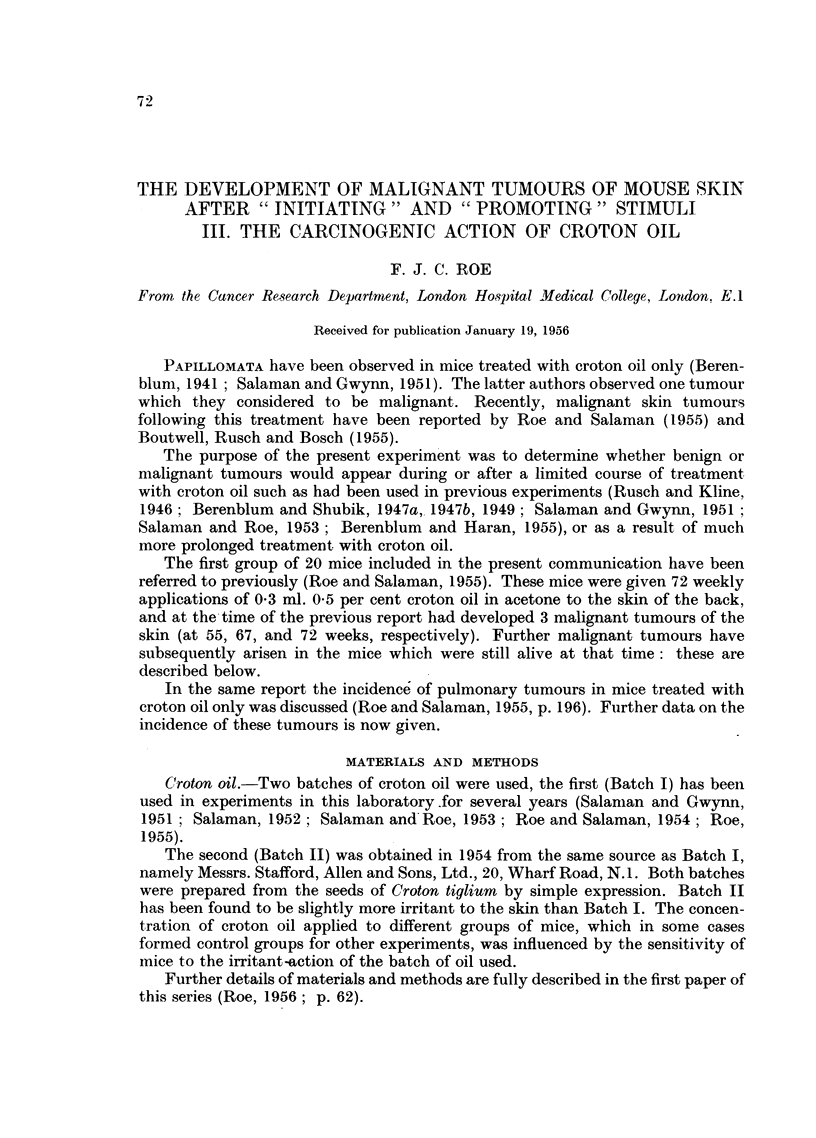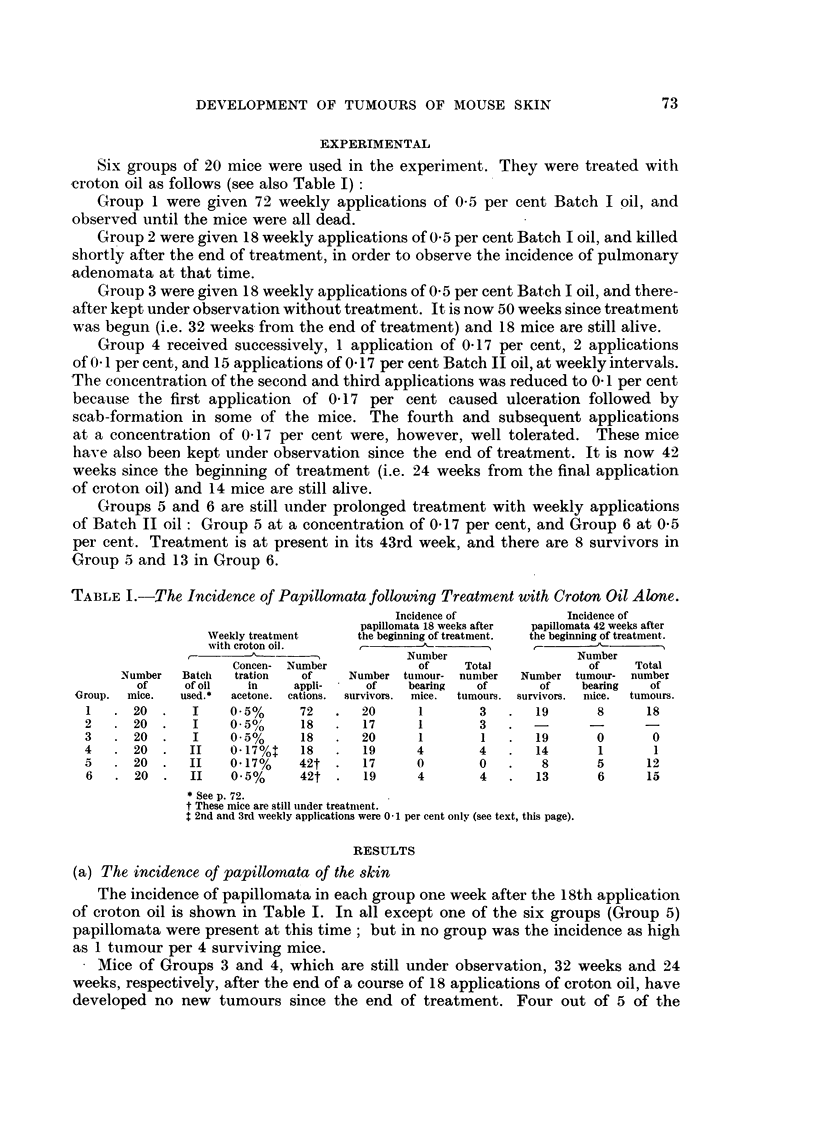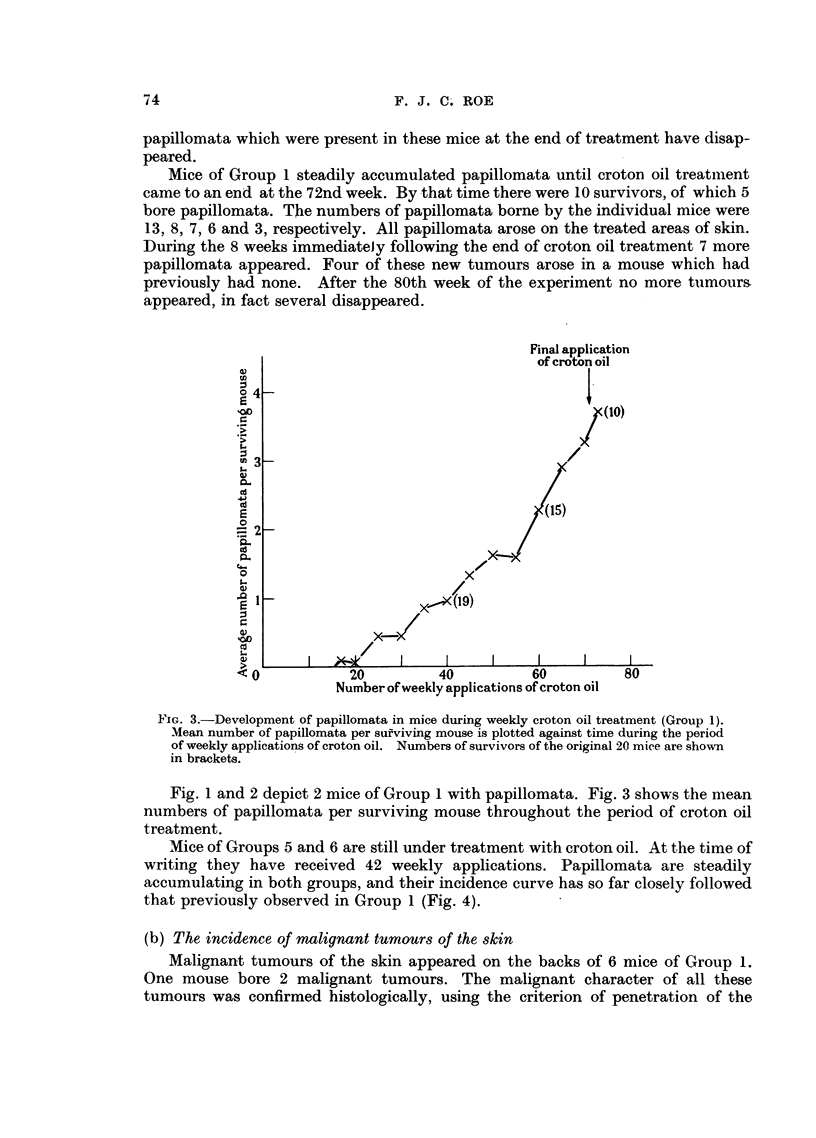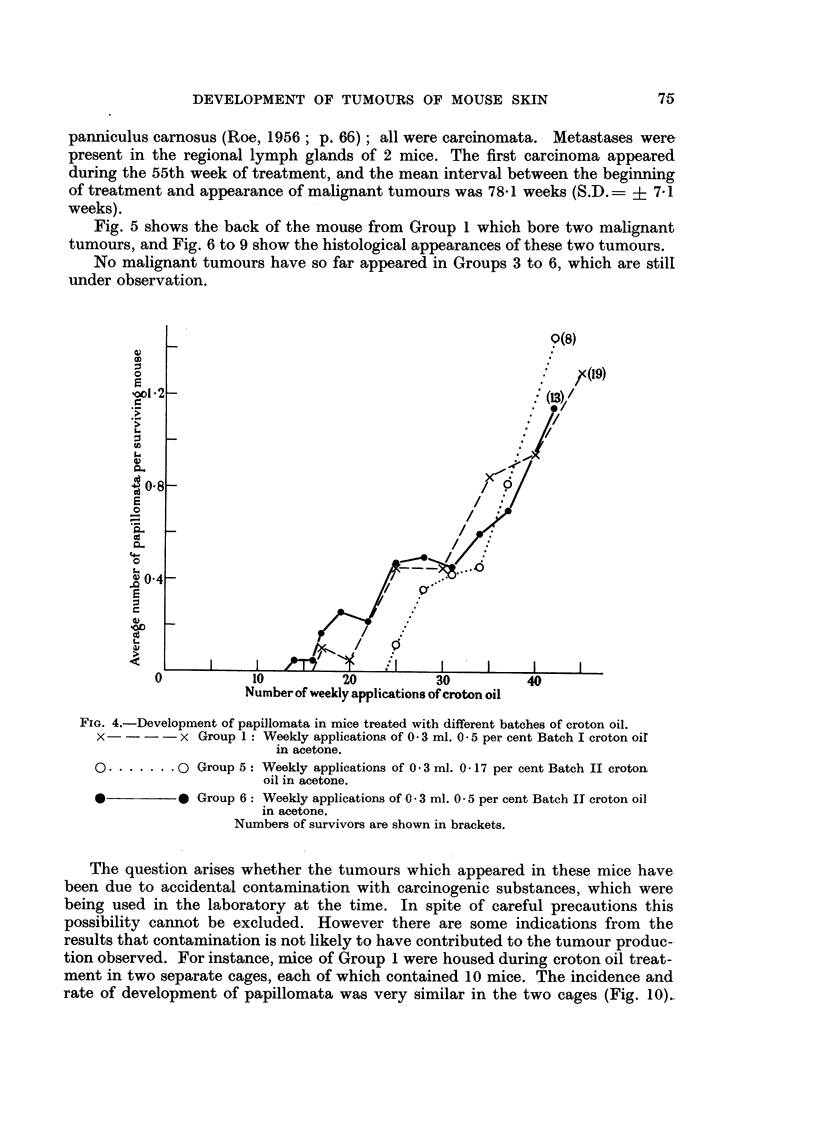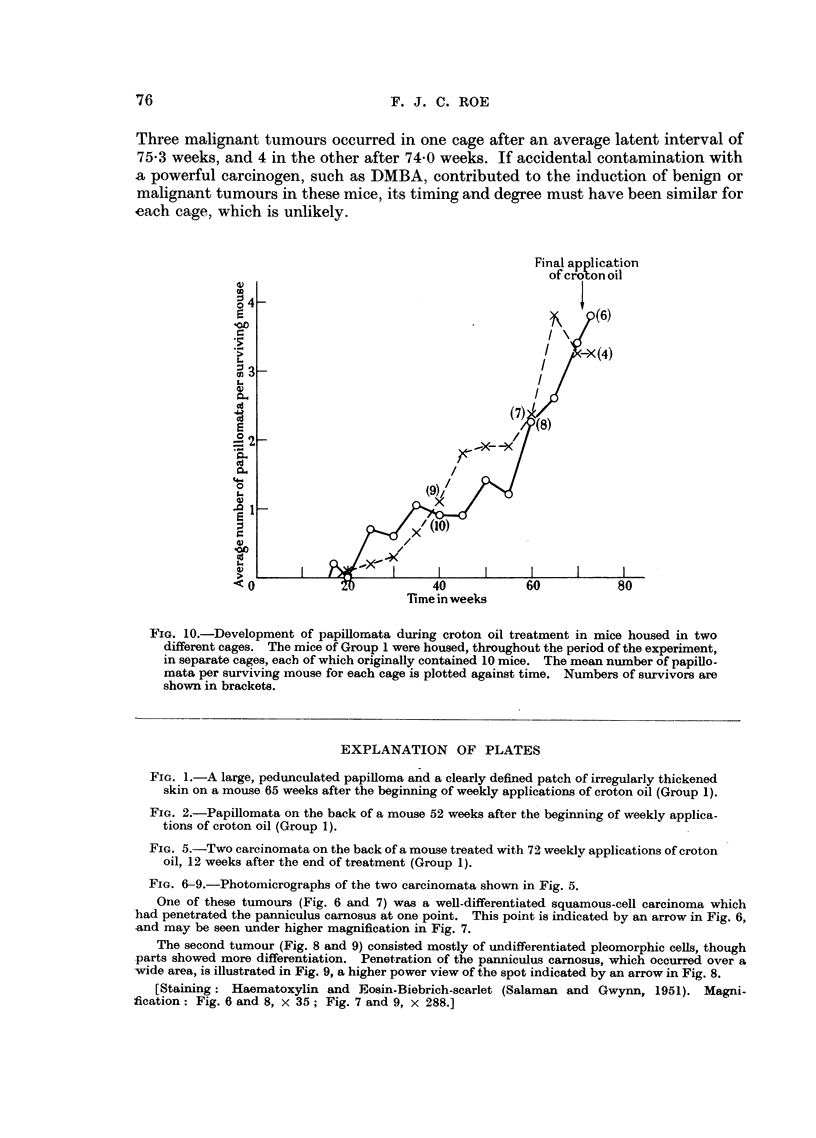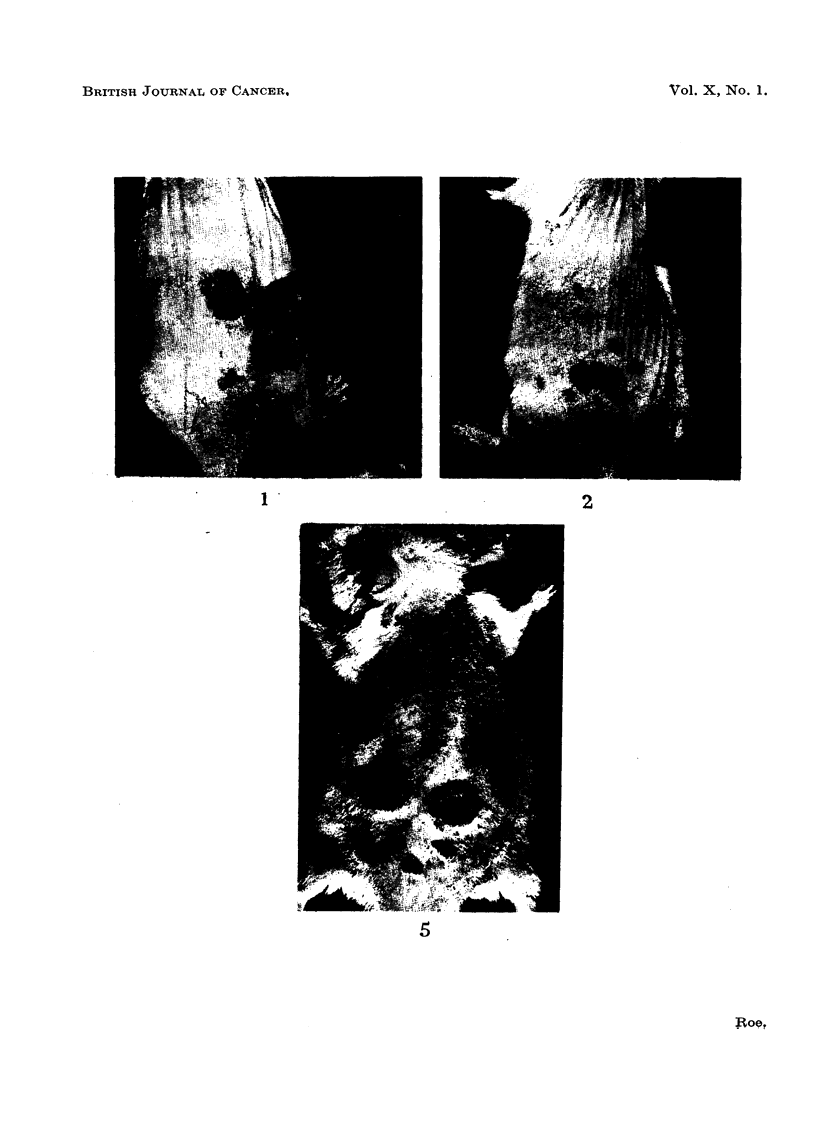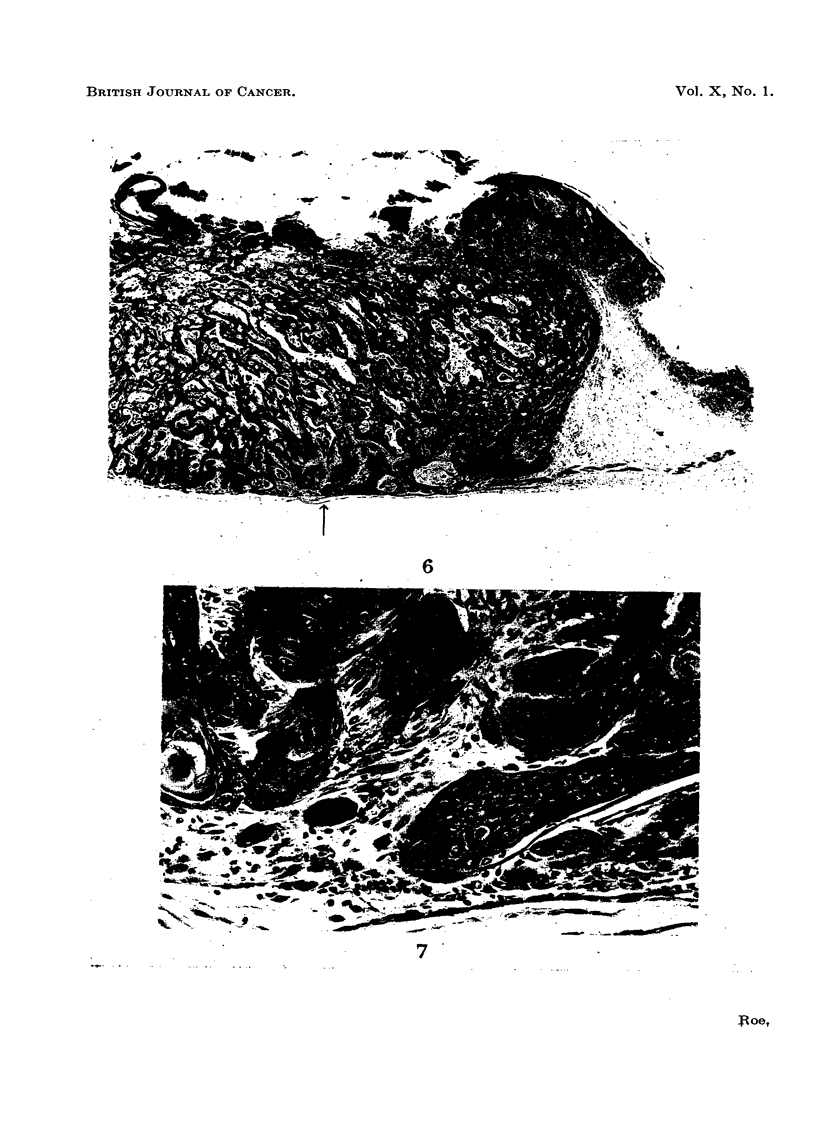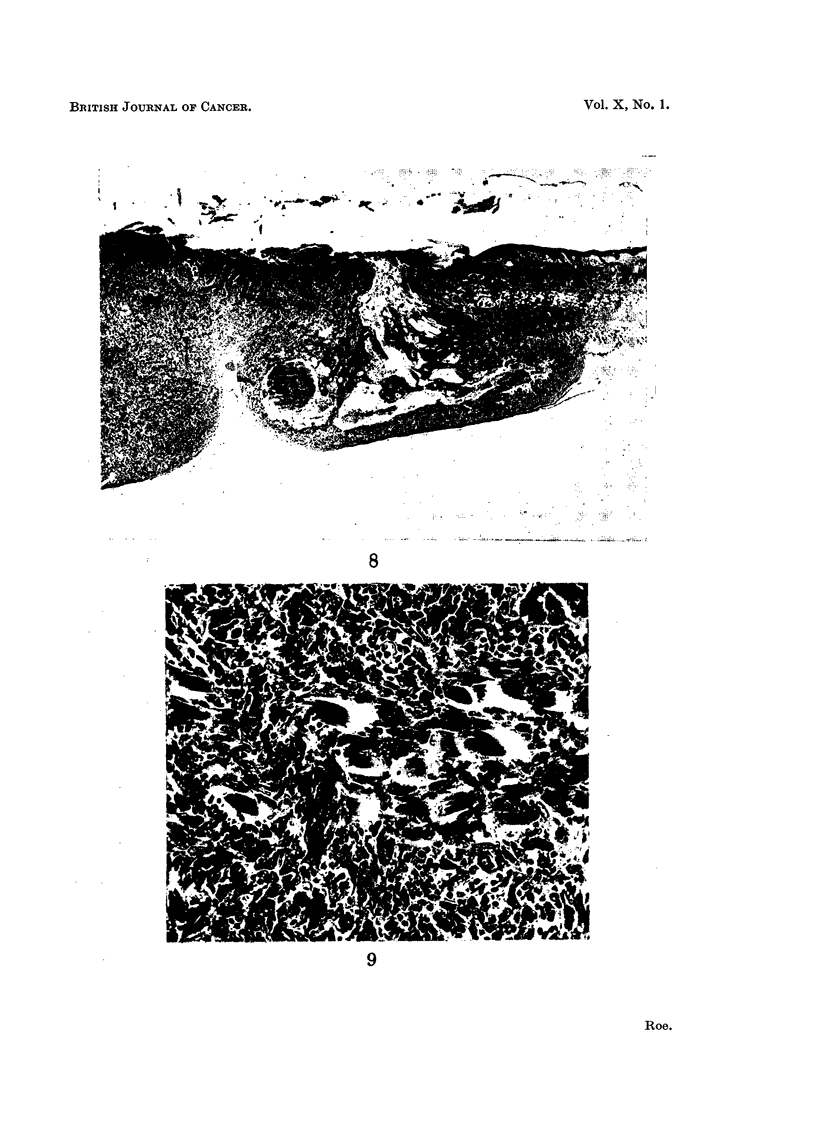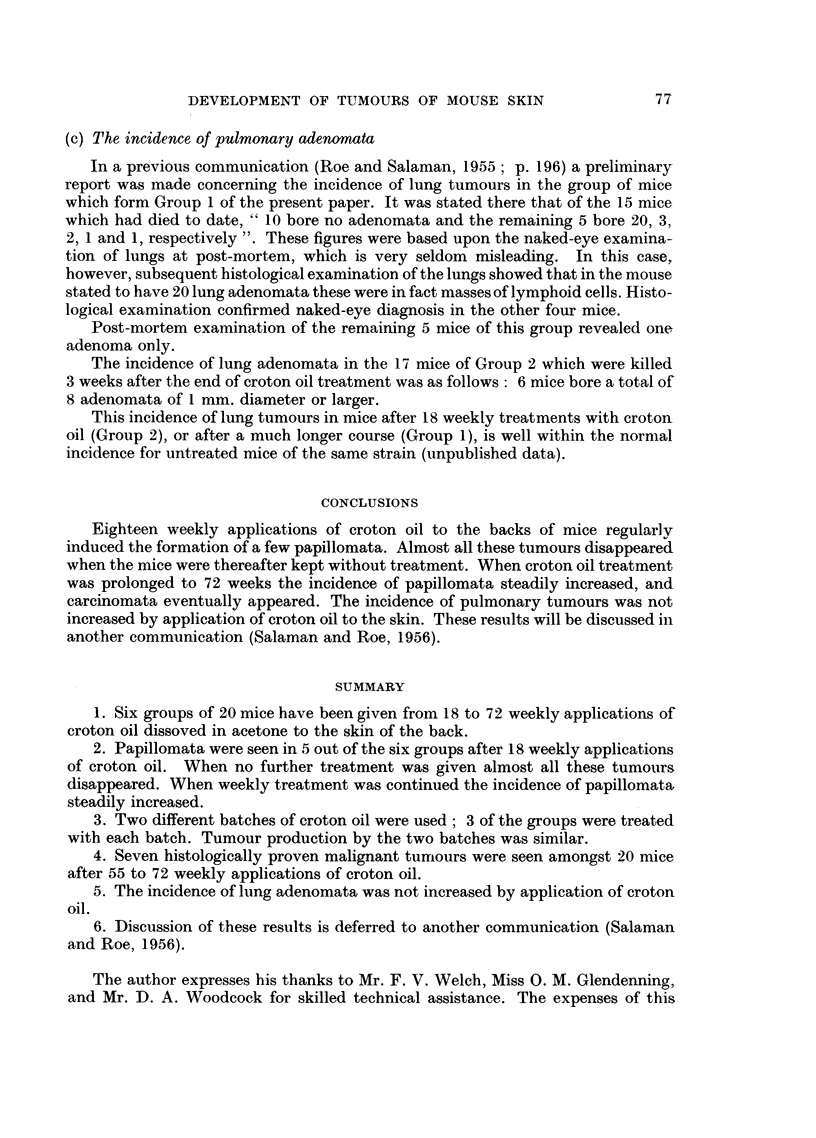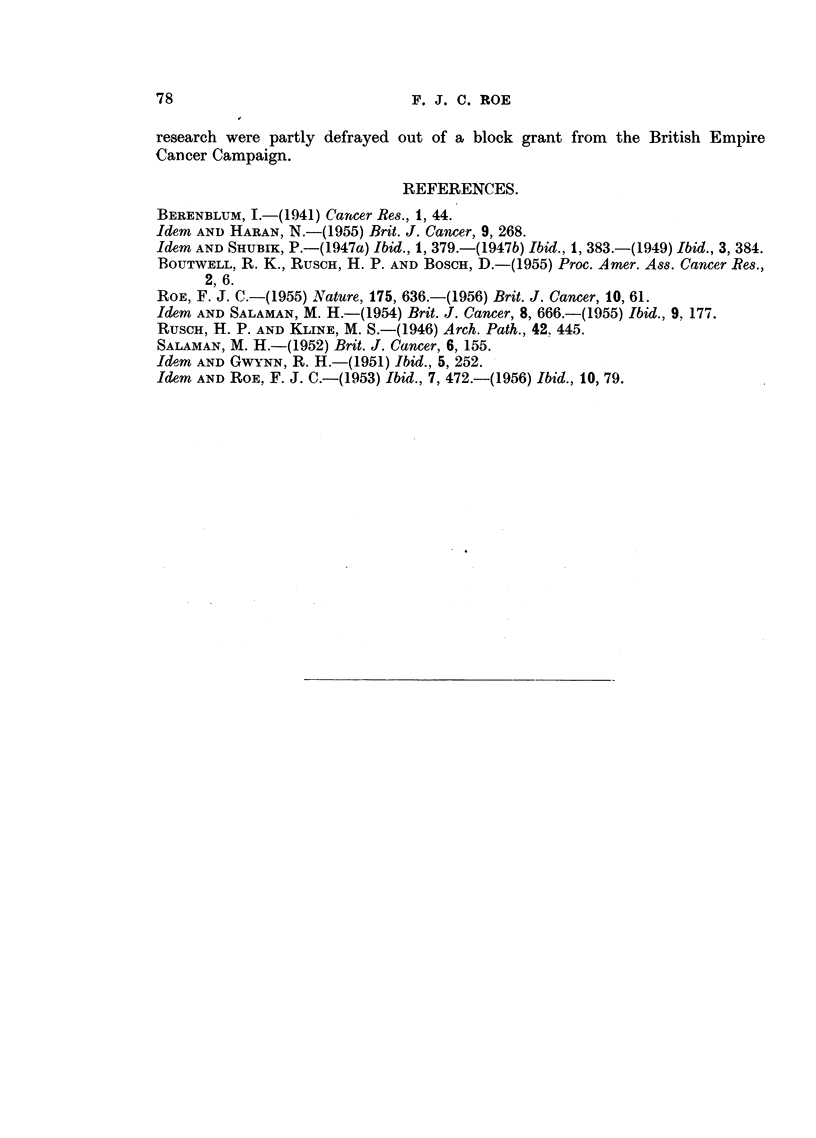# The Development of Malignant Tumours of Mouse Skin after “Initiating” and “Promoting” Stimuli. III. The Carcinogenic Action of Croton Oil

**DOI:** 10.1038/bjc.1956.11

**Published:** 1956-03

**Authors:** F. J. C. Roe

## Abstract

**Images:**


### THE DEVELOPMENT OF MALIGNANT TUMOURS OF MOUSE SKIN AFTER " INITIATING " AND " PROMOTING " STIMULI
### III. THE CARCINOGENIC ACTION OF CROTON OIL

F. J. C. ROE

*From the Cancer Research Department, London Hospital Medical College, London, E.*1

Received for publication January 19, 1956

PAPILLOMATA have been observed in mice treated with croton oil only (Berenblum, 1941 ; Salaman and Gwynn, 1951). The latter authors observed one tumour which they considered to be malignant. Recently, malignant skin tumours following this treatment have been reported by Roe and Salaman (1955) and Boutwell, Rusch and Bosch (1955).

The purpose of the present experiment was to determine whether benign or malignant tumours would appear during or after a limited course of treatment with croton oil such as had been used in previous experiments (Rusch and Kline, 1946 ; Berenblum and Shubik, 1947*a*, 1947*b*, 1949 ; Salaman and Gwynn, 1951 ; Salaman and Roe, 1953 ; Berenblum and Haran, 1955), or as a result of much more prolonged treatment with croton oil.

The first group of 20 mice included in the present communication have been referred to previously (Roe and Salaman, 1955). These mice were given 72 weekly applications of 0·3 ml. 0·5 per cent croton oil in acetone to the skin of the back, and at the time of the previous report had developed 3 malignant tumours of the skin (at 55, 67, and 72 weeks, respectively). Further malignant tumours have subsequently arisen in the mice which were still alive at that time : these are described below.

In the same report the incidence of pulmonary tumours in mice treated with croton oil only was discussed (Roe and Salaman, 1955, p. 196). Further data on the incidence of these tumours is now given.

### MATERIALS AND METHODS

*Croton oil.*—Two batches of croton oil were used, the first (Batch I) has been used in experiments in this laboratory for several years (Salaman and Gwynn, 1951 ; Salaman, 1952 ; Salaman and Roe, 1953 ; Roe and Salaman, 1954 ; Roe, 1955).

The second (Batch II) was obtained in 1954 from the same source as Batch I, namely Messrs. Stafford, Allen and Sons, Ltd., 20, Wharf Road, N.1. Both batches were prepared from the seeds of *Croton tiglium* by simple expression. Batch II has been found to be slightly more irritant to the skin than Batch I. The concentration of croton oil applied to different groups of mice, which in some cases formed control groups for other experiments, was influenced by the sensitivity of mice to the irritant action of the batch of oil used.

Further details of materials and methods are fully described in the first paper of this series (Roe, 1956 ; p. 62).

### EXPERIMENTAL

Six groups of 20 mice were used in the experiment. They were treated with croton oil as follows (see also Table I) :

Group 1 were given 72 weekly applications of 0·5 per cent Batch I oil, and observed until the mice were all dead.

Group 2 were given 18 weekly applications of 0·5 per cent Batch I oil, and killed shortly after the end of treatment, in order to observe the incidence of pulmonary adenomata at that time.

Group 3 were given 18 weekly applications of 0·5 per cent Batch I oil, and thereafter kept under observation without treatment. It is now 50 weeks since treatment was begun (i.e. 32 weeks from the end of treatment) and 18 mice are still alive.

Group 4 received successively, 1 application of 0·17 per cent, 2 applications of 0·1 per cent, and 15 applications of 0·17 per cent Batch II oil, at weekly intervals. The concentration of the second and third applications was reduced to 0·1 per cent because the first application of 0·17 per cent caused ulceration followed by scab-formation in some of the mice. The fourth and subsequent applications at a concentration of 0·17 per cent were, however, well tolerated. These mice have also been kept under observation since the end of treatment. It is now 42 weeks since the beginning of treatment (i.e. 24 weeks from the final application of croton oil) and 14 mice are still alive.

Groups 5 and 6 are still under prolonged treatment with weekly applications of Batch II oil : Group 5 at a concentration of 0·17 per cent, and Group 6 at 0·5 per cent. Treatment is at present in its 43rd week, and there are 8 survivors in Group 5 and 13 in Group 6.

TABLE I.—*The Incidence of Papillomata following Treatment with Croton Oil Alone.*

| Group. | Number of mice. | Weekly treatment with croton oil. Batch of oil used.* | Concentration in acetone. | Number of applications. | Incidence of papillomata 18 weeks after the beginning of treatment. Number of survivors. | Number of tumour-bearing mice. | Total number of tumours. | Incidence of papillomata 42 weeks after the beginning of treatment. Number of survivors. | Number of tumour-bearing mice. | Total number of tumours. |
|---|---|---|---|---|---|---|---|---|---|---|
| 1 | 20 | I | 0·5% | 72 | 20 | 1 | 3 | 19 | 8 | 18 |
| 2 | 20 | I | 0·5% | 18 | 17 | 1 | 3 | — | — | — |
| 3 | 20 | I | 0·5% | 18 | 20 | 1 | 1 | 19 | 0 | 0 |
| 4 | 20 | II | 0·17%‡ | 18 | 19 | 4 | 4 | 14 | 1 | 1 |
| 5 | 20 | II | 0·17% | 42† | 17 | 0 | 0 | 8 | 5 | 12 |
| 6 | 20 | II | 0·5% | 42† | 19 | 4 | 4 | 13 | 6 | 15 |

* See p. 72.
† These mice are still under treatment.
‡ 2nd and 3rd weekly applications were 0·1 per cent only (see text, this page).

### RESULTS

### (a) *The incidence of papillomata of the skin*

The incidence of papillomata in each group one week after the 18th application of croton oil is shown in Table I. In all except one of the six groups (Group 5) papillomata were present at this time ; but in no group was the incidence as high as 1 tumour per 4 surviving mice.

Mice of Groups 3 and 4, which are still under observation, 32 weeks and 24 weeks, respectively, after the end of a course of 18 applications of croton oil, have developed no new tumours since the end of treatment. Four out of 5 of the papillomata which were present in these mice at the end of treatment have disappeared.

Mice of Group 1 steadily accumulated papillomata until croton oil treatment came to an end at the 72nd week. By that time there were 10 survivors, of which 5 bore papillomata. The numbers of papillomata borne by the individual mice were 13, 8, 7, 6 and 3, respectively. All papillomata arose on the treated areas of skin. During the 8 weeks immediately following the end of croton oil treatment 7 more papillomata appeared. Four of these new tumours arose in a mouse which had previously had none. After the 80th week of the experiment no more tumours appeared, in fact several disappeared.

Fig. 3.—Development of papillomata in mice during weekly croton oil treatment (Group 1). Mean number of papillomata per surviving mouse is plotted against time during the period of weekly applications of croton oil. Numbers of survivors of the original 20 mice are shown in brackets.

Fig. 1 and 2 depict 2 mice of Group 1 with papillomata. Fig. 3 shows the mean numbers of papillomata per surviving mouse throughout the period of croton oil treatment.

Mice of Groups 5 and 6 are still under treatment with croton oil. At the time of writing they have received 42 weekly applications. Papillomata are steadily accumulating in both groups, and their incidence curve has so far closely followed that previously observed in Group 1 (Fig. 4).

(b) *The incidence of malignant tumours of the skin*

Malignant tumours of the skin appeared on the backs of 6 mice of Group 1. One mouse bore 2 malignant tumours. The malignant character of all these tumours was confirmed histologically, using the criterion of penetration of the panniculus carnosus (Roe, 1956 ; p. 66) ; all were carcinomata. Metastases were present in the regional lymph glands of 2 mice. The first carcinoma appeared during the 55th week of treatment, and the mean interval between the beginning of treatment and appearance of malignant tumours was 78·1 weeks (S.D. = $\pm$ 7·1 weeks).

Fig. 5 shows the back of the mouse from Group 1 which bore two malignant tumours, and Fig. 6 to 9 show the histological appearances of these two tumours.

No malignant tumours have so far appeared in Groups 3 to 6, which are still under observation.

FIG. 4.—Development of papillomata in mice treated with different batches of croton oil.

×— — — — —× Group 1 : Weekly applications of 0·3 ml. 0·5 per cent Batch I croton oil in acetone.

○· · · · · · ·○ Group 5 : Weekly applications of 0·3 ml. 0·17 per cent Batch II croton oil in acetone.

●————● Group 6 : Weekly applications of 0·3 ml. 0·5 per cent Batch II croton oil in acetone.

Numbers of survivors are shown in brackets.

The question arises whether the tumours which appeared in these mice have been due to accidental contamination with carcinogenic substances, which were being used in the laboratory at the time. In spite of careful precautions this possibility cannot be excluded. However there are some indications from the results that contamination is not likely to have contributed to the tumour production observed. For instance, mice of Group 1 were housed during croton oil treatment in two separate cages, each of which contained 10 mice. The incidence and rate of development of papillomata was very similar in the two cages (Fig. 10).

Three malignant tumours occurred in one cage after an average latent interval of 75·3 weeks, and 4 in the other after 74·0 weeks. If accidental contamination with a powerful carcinogen, such as DMBA, contributed to the induction of benign or malignant tumours in these mice, its timing and degree must have been similar for each cage, which is unlikely.

Fig. 10.—Development of papillomata during croton oil treatment in mice housed in two different cages. The mice of Group 1 were housed, throughout the period of the experiment, in separate cages, each of which originally contained 10 mice. The mean number of papillomata per surviving mouse for each cage is plotted against time. Numbers of survivors are shown in brackets.

---

### EXPLANATION OF PLATES

Fig. 1.—A large, pedunculated papilloma and a clearly defined patch of irregularly thickened skin on a mouse 65 weeks after the beginning of weekly applications of croton oil (Group 1).

Fig. 2.—Papillomata on the back of a mouse 52 weeks after the beginning of weekly applications of croton oil (Group 1).

Fig. 5.—Two carcinomata on the back of a mouse treated with 72 weekly applications of croton oil, 12 weeks after the end of treatment (Group 1).

Fig. 6–9.—Photomicrographs of the two carcinomata shown in Fig. 5.

One of these tumours (Fig. 6 and 7) was a well-differentiated squamous-cell carcinoma which had penetrated the panniculus carnosus at one point. This point is indicated by an arrow in Fig. 6, and may be seen under higher magnification in Fig. 7.

The second tumour (Fig. 8 and 9) consisted mostly of undifferentiated pleomorphic cells, though parts showed more differentiation. Penetration of the panniculus carnosus, which occurred over a wide area, is illustrated in Fig. 9, a higher power view of the spot indicated by an arrow in Fig. 8.

[Staining : Haematoxylin and Eosin-Biebrich-scarlet (Salaman and Gwynn, 1951). Magnification : Fig. 6 and 8, × 35 ; Fig. 7 and 9, × 288.]

1

2

5

6

7

8

9

(c) *The incidence of pulmonary adenomata*

In a previous communication (Roe and Salaman, 1955 ; p. 196) a preliminary report was made concerning the incidence of lung tumours in the group of mice which form Group 1 of the present paper. It was stated there that of the 15 mice which had died to date, " 10 bore no adenomata and the remaining 5 bore 20, 3, 2, 1 and 1, respectively ". These figures were based upon the naked-eye examination of lungs at post-mortem, which is very seldom misleading. In this case, however, subsequent histological examination of the lungs showed that in the mouse stated to have 20 lung adenomata these were in fact masses of lymphoid cells. Histological examination confirmed naked-eye diagnosis in the other four mice.

Post-mortem examination of the remaining 5 mice of this group revealed one adenoma only.

The incidence of lung adenomata in the 17 mice of Group 2 which were killed 3 weeks after the end of croton oil treatment was as follows : 6 mice bore a total of 8 adenomata of 1 mm. diameter or larger.

This incidence of lung tumours in mice after 18 weekly treatments with croton oil (Group 2), or after a much longer course (Group 1), is well within the normal incidence for untreated mice of the same strain (unpublished data).

### CONCLUSIONS

Eighteen weekly applications of croton oil to the backs of mice regularly induced the formation of a few papillomata. Almost all these tumours disappeared when the mice were thereafter kept without treatment. When croton oil treatment was prolonged to 72 weeks the incidence of papillomata steadily increased, and carcinomata eventually appeared. The incidence of pulmonary tumours was not increased by application of croton oil to the skin. These results will be discussed in another communication (Salaman and Roe, 1956).

### SUMMARY

1. Six groups of 20 mice have been given from 18 to 72 weekly applications of croton oil dissoved in acetone to the skin of the back.

2. Papillomata were seen in 5 out of the six groups after 18 weekly applications of croton oil. When no further treatment was given almost all these tumours disappeared. When weekly treatment was continued the incidence of papillomata steadily increased.

3. Two different batches of croton oil were used ; 3 of the groups were treated with each batch. Tumour production by the two batches was similar.

4. Seven histologically proven malignant tumours were seen amongst 20 mice after 55 to 72 weekly applications of croton oil.

5. The incidence of lung adenomata was not increased by application of croton oil.

6. Discussion of these results is deferred to another communication (Salaman and Roe, 1956).

The author expresses his thanks to Mr. F. V. Welch, Miss O. M. Glendenning, and Mr. D. A. Woodcock for skilled technical assistance. The expenses of this research were partly defrayed out of a block grant from the British Empire Cancer Campaign.

### REFERENCES.

BERENBLUM, I.—(1941) *Cancer Res.*, **1**, 44.
*Idem* AND HARAN, N.—(1955) *Brit. J. Cancer*, **9**, 268.
*Idem* AND SHUBIK, P.—(1947*a*) *Ibid.*, **1**, 379.—(1947*b*) *Ibid.*, **1**, 383.—(1949) *Ibid.*, **3**, 384.
BOUTWELL, R. K., RUSCH, H. P. AND BOSCH, D.—(1955) *Proc. Amer. Ass. Cancer Res.*, **2**, 6.
ROE, F. J. C.—(1955) *Nature*, **175**, 636.—(1956) *Brit. J. Cancer*, **10**, 61.
*Idem* AND SALAMAN, M. H.—(1954) *Brit. J. Cancer*, **8**, 666.—(1955) *Ibid.*, **9**, 177.
RUSCH, H. P. AND KLINE, M. S.—(1946) *Arch. Path.*, **42**, 445.
SALAMAN, M. H.—(1952) *Brit. J. Cancer*, **6**, 155.
*Idem* AND GWYNN, R. H.—(1951) *Ibid.*, **5**, 252.
*Idem* AND ROE, F. J. C.—(1953) *Ibid.*, **7**, 472.—(1956) *Ibid.*, **10**, 79.